# Effect of a Diet Supplemented with Sphingomyelin and Probiotics on Colon Cancer Development in Mice

**DOI:** 10.1007/s12602-022-09916-6

**Published:** 2022-02-02

**Authors:** Florencio Marzo, Patricia Jauregui, Jaione Barrenetxe, Ana Martínez-Peñuela, Francisco C. Ibañez, Fermin I. Milagro

**Affiliations:** 1 Laboratory of Animal Physiology and Nutrition, School of Agronomy, Universidad Pública de Navarra, Pamplona, Spain; 2 Department of Nutrition, Food Science and Physiology, Center for Nutrition Research, University of Navarra, Pamplona, Spain; 3 Laboratorio Martínez-Peñuela, Pamplona, Spain; 4 Navarra Institute for Health Research (IdISNA), Pamplona, Spain; 5 Centro de Investigación Biomédica en Red de La Fisiopatología de La Obesidad Y Nutrición (CIBERobn), Instituto de Salud Carlos III, Madrid, Spain

**Keywords:** Sphingomyelin, Aberrant crypt foci, 1,2-Dimethylhydrazine, *Lacticaseibacillus casei*, *Bifidobacterium bifidum*

## Abstract

Previous studies have reported that dietary sphingomyelin could inhibit early stages of colon cancer. Lactic acid–producing bacteria have also been associated with an amelioration of cancer symptoms. However, little is known about the potential beneficial effects of the combined administration of both sphingomyelin and lactic acid–producing bacteria. This article analyzes the effect of a diet supplemented with a combination of the probiotics *Lacticaseibacillus casei* and *Bifidobacterium bifidum* (10^8^ CFU/ml) and sphingomyelin (0.05%) on mice with 1,2-dimethylhydrazine (DMH)-induced colon cancer. Thirty-six BALB/c mice were divided into 3 groups: one healthy group (group C) and two groups with DMH-induced cancer, one fed a standard diet (group D) and the other fed a diet supplemented with sphingomyelin and probiotics (DS). The number of aberrant crypt foci, marker of colon cancer development, was lower in the DS. The dietary supplementation with the synbiotic reversed the cancer-induced impairment of galactose uptake in enterocyte brush–border–membrane vesicles. These results confirm the beneficial effects of the synbiotic on the intestinal physiology of colon cancer mice and contribute to the understanding of the possible mechanisms involved.

## Introduction

Colorectal cancer (CRC) is the third most common diagnosed cancer representing an important health issue worldwide. Despite strong hereditary components, about 80% of cases of colorectal cancers are sporadic and develop slowly over more than 10 years. Epidemiologic studies have shown that the environmental factors, especially diet, are important risk factors for CRC [1, 2]

Mounting evidence supports the view that the colonic microbiota is involved in the etiology of colon cancer. Different factors, such as probiotics and different dietary bioactive compounds can modulate gut microbiota and its metabolism [3]. In this context, several investigations have focused on the beneficial effects of probiotics and bioactive compounds and their possible role in the prevention of colon cancer [4, 5]. Most probiotics are members of the genus *Bifidobacterium* and several genera of the *Lactobacillus* group, but *Saccharomyces* and *Enterococcus* have also been studied. Experimental animal and human studies have shown that probiotics may reduce intestinal permeability and participate in the regulation of several intestinal functions [6–8]. Concretely, animal studies have demonstrated that certain species of lactic acid–producing bacteria, such as *Lacticaseibacillus casei* [9] and *Bifidobacterium bifidum* [10], could prevent colon cancer and other diseases linked to the gastrointestinal tract.

On the other hand, sphingolipids are structural and functional bioactive lipids found in eggs, milk, meat, fish, and soybeans that can act as chemo-protective agents regulating cell growth, differentiation, and death [11, 12]. Sphingomyelin (SM) is the most abundant sphingolipid in plasma lipoproteins and contains predominantly a phosphocholine as head group [13]. The hydrolysis of SM by alkaline sphingomyelinase (alk-SMase) generates other bioactive molecules, such as ceramide and sphingosine, that play key roles in the maintenance of intestinal mucosal integrity and the inhibition of colon tumorigenesis [12, 14]. Ceramide has been proposed as an important intracellular messenger in different signalling pathways implicated in the regulation of cellular proliferation, differentiation, and apoptosis [14–16]. Early studies have indicated that a therapy based on probiotics mixture containing several strains of acid lactic bacteria increased alk-SMase levels in mice with inflammatory gut disease [17] and cancer [18]. Furthermore, the combination of probiotic and other dietary compounds may exert additive effects in the improvement of colon carcinogenesis as compared to its separate administration [2, 19]. However, the combination of SM and probiotics has not yet been analyzed although it could be hypothesized that dietary supplementation with probiotics together with SM could help to maintain the membrane integrity of the enterocytes, as well as alk-SMase activity in colon cancer models. Therefore, in this study, we have analyzed, for the first time, the beneficial effects of the combined administration of both lactic acid–producing bacteria (*L. casei* and *B. bifidum*) and SM in a mouse model of 1,2-dimethylhydrazine (DMH)-induced colon cancer.

## Materials and Methods

### Bacterial Strains and Growth Condition

The *L. casei* CECT 475 T strain was isolated from kefir manufactured in the Lactology Laboratory of the Universidad Pública de Navarra, whereas the *B. bifidum* strain was obtained from the Spanish Collection of Cultures (CECT) with the number CECT 870. Both strains were used in this study as probiotics. Both bacteria were grown in autoclaved skim milk (Difco™; BD, Detroit, MI, USA), at 37 °C for 24 h under aerobic conditions for *L. casei* and at 39 °C for 72 h under microaerophilic conditions for *B. bifidum* in the presence of 5% CO_2_. The cell pellets were resuspended in 10% Difco™ skim milk at a final concentration of 10^8^ CFU/ml for each strain.

### Chemicals

All chemicals were purchased from Sigma Chemicals (St. Louis, MO, USA) unless otherwise noted. All reagents were of analytical grade.

### Animals, Diets, and Experimental Design

Thirty-six male BALB/c mice of 28 days old and weighing about 20 g were obtained from the colony of Charles River Laboratory Animals (Barcelona, Spain). The mice were housed in cages (four animals per cage) and kept in a well-ventilated, thermostatically controlled room (22 ± 2 °C temperature and 55 ± 5% relative humidity) with a photoperiod of 12 h light/night cycle.

The animals, after an acclimatization period of 4 days, were weighed and randomly assigned to 3 homogeneous groups (*n* = 12, each) and fed a standard diet (AIN-93G, Research Diets Inc., New Brunswick, NJ, USA). Control group (C) animals were injected subcutaneously with ethylenediaminetetraacetic acid (EDTA) 1 mM, whereas the mice of the DMH group (D) and DMH + supplemented diet group (DS) were injected subcutaneously with 1,2-dimethylhydrazine (DMH) dissolved in EDTA 1 mM (30 mg DMH/kg body weight, twice per week for 3 weeks) to induce the pre-neoplastic lesions (ACF).

Fresh diets were prepared weekly with purified ingredients. All diets were isonitrogenous and isoenergetic and were stored at 4 °C until serving feed. One week after the last DMH injection, the animals in the DS group were fed the standard diet supplemented with sphingomyelin 0.05% and skim milk was supplemented with probiotic bacteria at 10^8^ CFU/ml. Food and drink were available ad libitum. The body weight was recorded weekly.

At the end of the 66th day of the experimental period, the animals were anesthetized with CO_2_ and sacrificed by decapitation. Trunk blood was collected for the measurement of serum biochemical parameters, and different organs (spleen, liver, colon, jejunum, and cecum) were extracted and weighed. The jejunum was carefully removed, flushed out with ice-cold saline, frozen in liquid nitrogen and stored at −80 °C until processed for the isolation of the brush–border–membrane vesicles (BBMV) for the measurement of the intestinal absorption of D-galactose.

The Animal Research Ethics Committee of the “Universidad Pública de Navarra” reviewed and approved (reference PI:07/06) the animal care protocol and the killing method to ensure compliances with the guidelines of the Canadian Council on Animal Care [20].

### Histological Analyses

Colon sections of 6 animals per group were flushed with ice-cold saline, opened longitudinally, fixed flat in 10% formalin for 24 h, dehydrated, and stained in 0.1% methylene blue for 15 min. The colon pieces were placed mucosa-side up on glass slides and evaluated for the presence of aberrant crypts foci by light microscopy (40 × or 100 ×). The aberrant foci were identified following the counting criteria described by Paulsen et al. [21]. Before being frozen, a portion of the distal colon (1 cm length) was taken for histological examination. Samples of distal colon tissue were immediately fixed in 4% formalin solution for 24 h. They were then dehydrated, embedded in paraffin, sliced into 5-µm sections and processed for hematoxylin–eosin staining.

### Serum Biochemical Assays

All serologic parameters (Table 2) were quantified in an automatic chemistry analyzer Cobas-Mira (Roche Diagnostic System, Basel, Switzerland) following the manufacturer’s procedures.

### Preparation of Mouse Intestinal BBMV

BBMV were obtained according to the method described by Shirazi-Beechey et al. [22]. Briefly, the BBMV were prepared from a portion of small intestine extracted from each animal of the three different experimental groups.

The mucosa was then resuspended in buffer containing 100 mM mannitol and 2 mM 4-(2-hydroxyethyl)-1-piperazineethanesulfonic acid (HEPES) adjusted to pH 7.1 with 1 M Tris–HCl buffer. The suspension was homogenized with a Potter–Elvehjem (Braun, Melsungen, Germany) at 3000 rpm at 4 °C for 1 min. Next, MgCl_2_ was then added to the homogenate to a final concentration of 10 mM, and the mixture was maintained on ice with continuous ice-cold shaking for 20 min. After that, the mixture was centrifuged at 2000 g for 15 min, and the supernatant was collected and centrifuged at 27,000 g for 30 min. This supernatant was discarded and the pellet resuspended in a buffer containing 100 mM mannitol, 0·1 mM MgSO_4_ and 2 mM HEPES adjusted to pH 7·4 with Tris. After a second precipitation with MgCl_2_, the mixture was finally centrifuged at 27,000 g for 30 min. The pellet was then resuspended in a buffer containing 300 mM mannitol, 0·1 mM MgSO_4_ and 10 mM HEPES adjusted to pH 7·4 with Tris. The BBMV of ten mice were pooled, assayed for protein content by using the Bradford diagnostic kit (Bio-Rad Laboratories, Barcelona, Spain), diluted to 10 mg BBMV protein/ml, aliquoted and frozen in liquid nitrogen. The final BBMV preparation was fivefold enriched for sucrase specific activity compared with the initial homogenate.

### Sugar Uptake by BBMV

Sugar uptake by the BBMV was measured using a slightly modified version of the rapid filtration technique developed by Hopfer [23]. Three replications were performed for each experimental group. D-galactose (0.1 mM) uptake was determined in the presence of a Na^+^ gradient at pH 7.4 and 37 °C. BBMV were incubated in a medium containing 0.1 mM D-galactose, 100 mM NaSCN, 100 mM mannitol, 0.1 mM MgSO_4_, 10 mM HEPES, and traces of D-[1-^14^C]galactose (0.037 MBq/ml; Amersham Radiochemical Centre, UK). At the different incubation times, uptake was halted by adding ice-cold stop solution (150 mM KSCN, 0.25 mM phloridzin and 10 mM HEPES) at pH 7.4 for the galactose uptake determination.

The suspension was poured immediately onto a cellulose nitrate filter (0.45 μm, 25 mm diameter; Sartorius, Edgewood, NY, USA) and the filter was then washed twice in ice-cold stop solution and dissolved in HiSafe 3 scintillation liquid for the final measurement of radioactivity using a β-counter (1450 MicroBeta® TriLux; Wallac, Turku, Finland).

### Statistical Analysis

Descriptive and inferential statistics were used according to procedures described by Anderson [24]. The normality of the sample distribution of each continuous variable was tested with the Kolmogorov–Smirnov test. To identify significant differences among the groups (body and organ weights, and biochemical parameters), statistical analysis was performed by one-way ANOVA followed by Dunnett’s test (D group as reference). Kruskal–Wallis followed by Mann–Whitney *U* test (D group as reference) were used to compare Western blot data and the ACF number of the three experimental groups. Differences were considered statistically significant when 2-tailed *p* < 0.05. Statistical calculations were performed with the statistical software package SPSS version 21.0 for Windows (SPSS, Chicago, IL, USA).

## Results

### Animal Growth: Body and Organ Weights

**Fig. 1:**
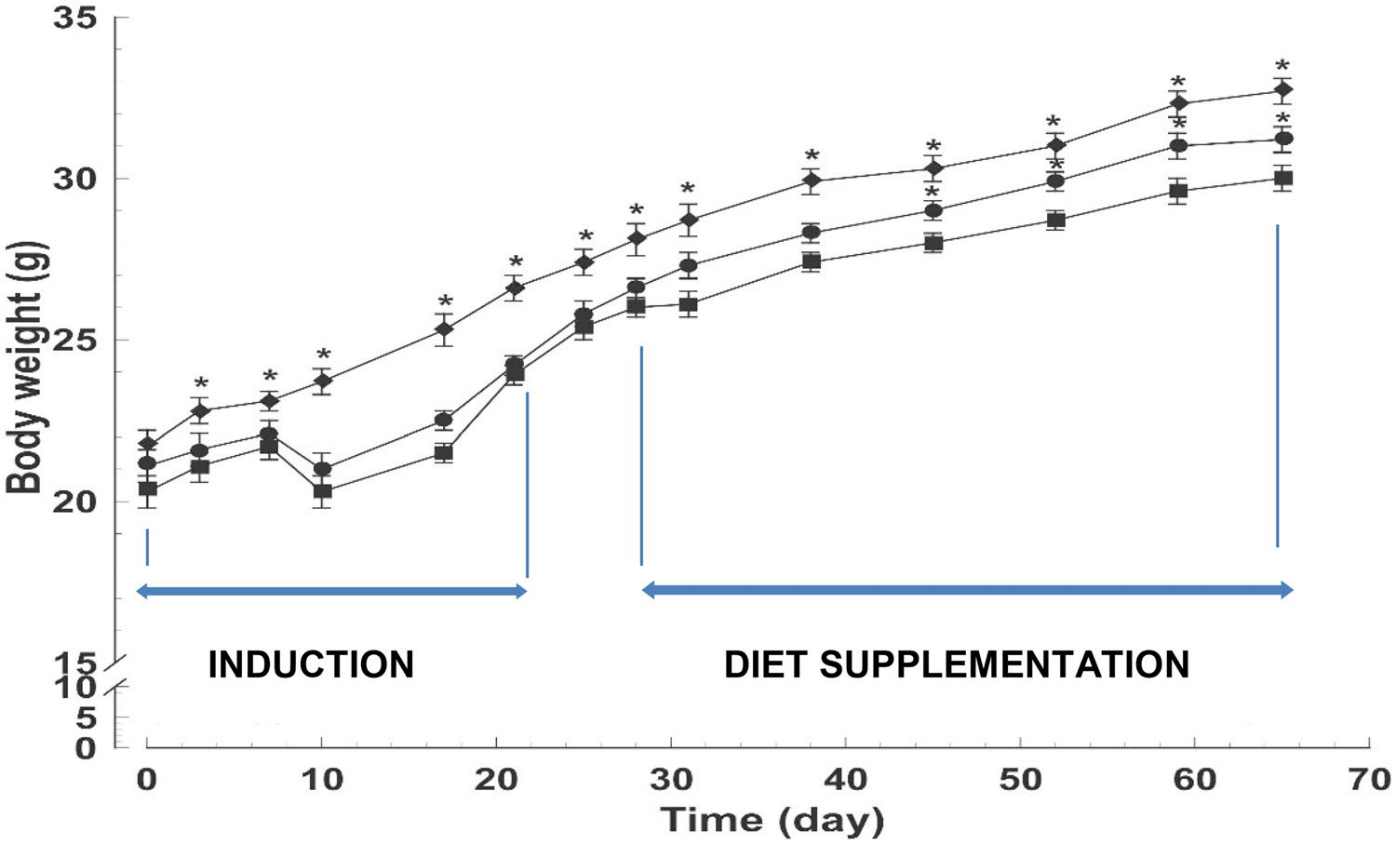
Body weight gain of the three experimental groups (*n* = 12 per group): filled diamond, control group; filled square, DMH group (D); filled circle, DMH + supplemented diet group (DS). Data are expressed as mean ± SEM. *p < 0.05 vs D group

The second week after cancer induction, body weight was significantly lower in the animals of the D and DS groups when compared with the C group (Fig. 1). The body weight of the animals of the DS group was significantly higher than those of the D group from day 45 of the experimental period, indicating that the dietary supplementation improves the growth rate of the animals.

Regarding the weights of the different tissues (Table 1), colon, and liver were the only organs that showed statistical differences when comparing the three experimental groups. Colon weight was higher in the DS group when compared with C (*p* = 0.002) and D (*p* = 0.015) groups, whereas liver weight was lower in D (*p* = 0.001) and DS (*p* = 0.007) groups when compared with C group.

**Table 1:** Organ weights of the three experimental groups

Experimental groups	C	D	DS
Jejunum (g)	0.724 ± 0.011	0.677 ± 0.025	0.706 ± 0.048
Colon (g)	0.163 ± 0.101	0.171 ± 0.007	0.199 ± 0.006*^/^**
Liver (g)	1.639 ± 0.045	1.434 ± 0.031*	1.488 ± 0.033*

Data (*n* = 12 per group) are expressed as mean ± SEM

*C* control group, *D* DMH group, *DS* DMH + supplemented diet group

^*^p <0.01 vs C group; **p <0.05 vs D group (highlighted in bold font)

### Serum Biochemical Measurements

Serum glucose, urea, total cholesterol and high-density lipoprotein cholesterol levels tended to be higher in the D and DS groups, but without statistical differences (Table 2). Serum aspartate aminotransferase levels were significantly lower in the control group and in the DS group when compared to D group although statistically significant differences were only observed in control group. In this line, alanine aminotransferase levels were also significantly lower in control and DS groups when compared to D group indicating that the induction of pre-neoplastic lesions in the colon increased the enzymatic activity and that the administration of the symbiotic combination reduced this increase, although control levels were not reached. This altered enzymatic activity observed in the DMH treated animals confirms the DMH-induced liver damage observed in treated animals. The supplementation with SM and probiotics did not prevent the liver weight loss observed in the DMH-treated mice, but it reverted the increase in alanine amino transferase serum level (*p* ≤ 0.05).

**Table 2:** Serum biochemical parameters of the experimental groups

Experimental groups	C	D	DS
Glucose (mg/dl)	86.4 ± 5.8	104.2 ± 5.9	97.9 ± 5.3
Urea (mg/dl)	38.8 ± 4.9	45.7 ± 3.7	48.1 ± 5.2
Plasma protein (g/dl)	3.23 ± 0.18	2.91 ± 0.17	2.85 ± 0.16
Albumin (g/dl)	2.13 ± 0.21	1.94 ± 0.20	2.05 ± 0.13
Aspartate aminotransferase (U/l)	68.6 ± 5.0*	109.5 ± 9.7	86.5 ± 8.7
Alanine aminotransferase (U/l)	16.1 ± 1.8*	40.7 ± 3.9	30.3 ± 3.2*
Lactate dehydrogenase (U/l)	553.8 ± 58.1	577.5 ± 83.0	624.0 ± 54.0
α-amylase (U/l)	3773 ± 416	4091 ± 430	4522 ± 373
Cholesterol (mg/dl)	76.5 ± 6.3	83.8 ± 7.9	75.0 ± 5.5
HDL-cholesterol (mg/dl)	61.3 ± 5.6	64.5 ± 7.0	61.6 ± 3.7
Cholesterol:HDL cholesterol ratio	1.28 ± 0.04	1.34 ± 0.05	1.22 ± 0.05

Data (n = 12 per group) are expressed as mean ± SEM

*C* control, *D* DMH group, *DS* DMH + supplemented diet group

^*^p < 0.05 vs D group (highlighted in bold font)

### Histological Analysis

**Fig. 2:**
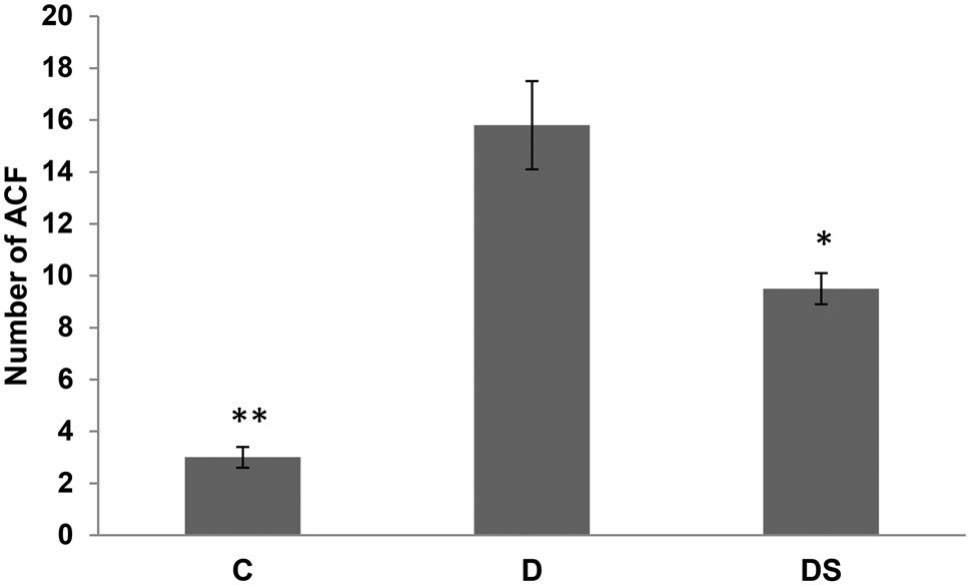
Number of aberrant crypt foci (ACF) in control (C), DMH group (D) and DMH + supplemented diet group (DS). Data are expressed as mean ± SEM (*n* = 4). *p < 0.05 vs D group: **p < 0.01 vs D group

**Fig. 3:**
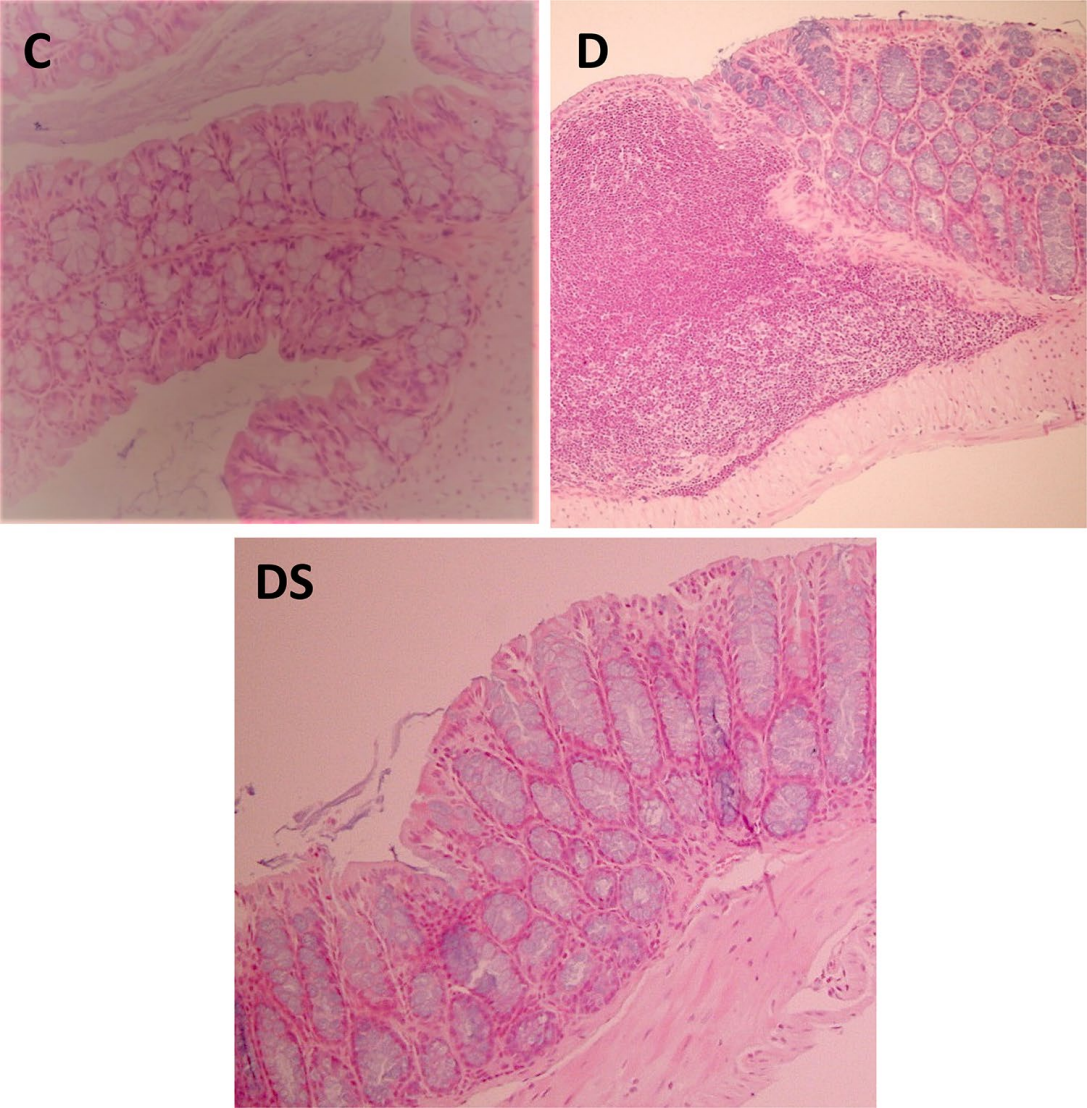
Hematoxylin–eosin staining of the colon obtained from the different experimental groups (× 40). *C*, control group; D, DMH group; DS, DMH + supplemented diet group

These ACF are considered indicators of pre-neoplastic damage. All the ACF found were located in the distal colon. Interestingly, dietary supplementation with sphingomyelin plus *L. casei and B. bifidum* resulted in a significantly reduction in the number of ACF when compared with the D group (Fig. 2). Colon sections of control mice displayed normal crypt foci and a colonic architecture with no signs of apparent abnormality. However, large areas with dense lymphocytic hyperplasia, compatible with pre-neoplastic lesions, were observed in the D group. Interestingly, this high lymphocyte infiltration was reduced by the administration of the symbiotic compound (Fig. 3).

### Sugar Uptake in BBMV

**Fig. 4:**
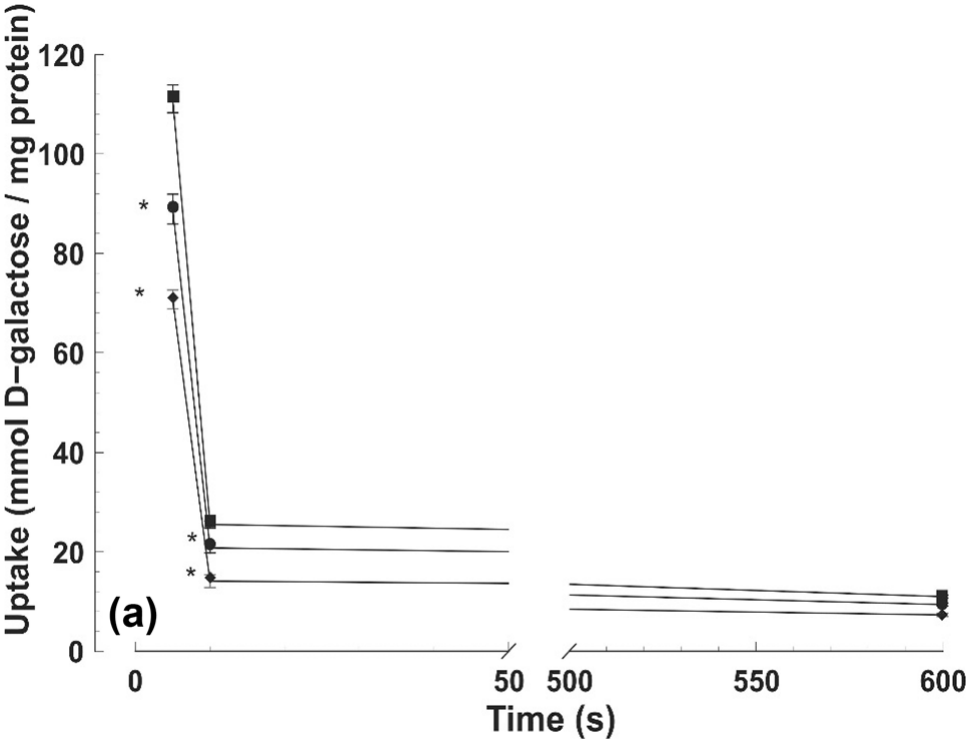
Uptake of 1 mM D-galactose by intestinal BBMV obtained from mice (*n* = 3 per group): filled diamond, control group (C); filled square, DMH group (D); filled circle, DMH + supplemented diet group (DS). Data are expressed as mean ± SEM. *p < 0.05 vs D group

Intestinal function has also been analyzed in the present work by measuring D-galactose uptake in BBMV. In this context, sugar uptake was stimulated by CRC development. The presence of the synbiotic in the diet inhibited D-galactose uptake as compared to DMH group (Fig. 4). This effect was observed only when the nutrients entered the enterocyte by an active transport mechanism that was mediated by a transporter located in the brush–border–membrane (short assay times and a Na^+^ gradient). For longer incubation times (10 min and 60 min, respectively), the sugar entered the vesicles by a process of diffusion that was not altered by the synbiotic.

## Discussion

Previous reports have demonstrated that several probiotics and bioactive compounds have a positive effect on the immune system, improving the immune response by unspecific and specific mechanisms [25]. In the context of colorectal cancer, the protective effect of dietary supplementation with different probiotics and synbiotics has been studied in animal models [26]. The present work has analyzed the effects of the combination of two probiotics (*L. casei*, *B. bifidum*) and a sphingolipid (sphingomyelin) on the physiopathology and intestinal function of mice with DMH-induced colon cancer. As there are no animal models that develop colon cancer spontaneously, DMH has been used to induce pre-cancerous lesions that are histologically similar to those observed in humans and has been widely used in the literature [27, 28].

Regarding body weight, the results obtained are in agreement with other studies that did not find differences in body weight gain after supplementation with SM [11, 15] or probiotics [6] and in line with studies by other authors in which DMH has been used as carcinogen [29]. Although sphingolipids have relevant biological activities, they are not considered as essential nutrients for an optimal growth rate of the animals [30, 31].

As it has been previously mentioned, colon and liver were the only organs affected by the pre-cancerous lesion induction or synbiotic treatment. DMH-treated animals (D) showed a tendency to a higher colon weight than the untreated control group. This effect could be due to the initial stage of the disease that occurs with the presence of colon mucosa polyps that would increase the organ weight. However, the colon of the animals treated with the supplemented diet (DS group) was significantly higher than the one of the DMH group (D) suggesting that the consumed bacteria might attach to the colonic mucosae and increase, in consequence, the colon weight. In this sense, other authors have demonstrated that some strains of the *Lactobacillus* group are able to colonize the colon and adhere to the intestinal epithelium [32]. On the other hand, the liver was also affected by CRC development. Liver weight was lower in the animals with DMH-induced cancer than in the control group, suggesting that liver damage is related to colon cancer development and therefore could be implicated in the body weight loss observed in these animals. This liver damage was confirmed by the altered levels of serum alanine aminotransferase and aspartate aminotransferase observed in D and DS groups. In this context, a previous study found lower liver weight and decreased hepatic lipid accumulation in mice fed a diet supplemented with 0.2–0.4% sphingolipids [33]. Furthermore, other authors have shown that treatment with probiotics like *Lactobacilli* and *Bifidobacteria* could improve liver function [34].

Moreover, the dietary supplementation decreased the number of ACF as compared to D group, as found in previous studies with flavonoids [35]. Similar to our results, Li et al. demonstrated that germinated brown rice grains fermented by *Lactobacillus acidophilus* could modulate the formation of pre-neoplastic lesions through the activation of apoptotic pathways [36]. Benito et al. have reported that microencapsulated *B. bifidum* and *Lactobacillus gasseri* can inhibit CRC development in Apc^Min/+^ mice [37]. Finally, the inhibition of tumor formation due to dietary sphingomyelin has been attributed to a normalization of cell proliferation and rate of apoptosis, but not the induction of differentiation [12].

Finally, it seems that diet supplementation with the two bacteria and sphingomyelin inhibits the mechanisms involved in sugar absorption stimulation observed in DMH animals, restoring the sugar uptake levels obtained in control mice. These findings are in line with previous studies that demonstrated that probiotics [6] and sphingomyelin [11] are able to decrease glucose transport in intestinal epithelial cells models. These results suggest that the administration of the synbiotic could help to prevent colon cancer development in humans since the stimulation of sugar uptake increases the amount of intracellular glucose available for metabolic conversion, thereby promoting enhanced cell proliferation and cancer development [38].

In summary, the dietary supplementation with the synbiotic preparation reduced the number of histological intestinal lesions, suggesting that this combination has beneficial effects in DMH-treated mice with pre-neoplastic lesions. Moreover, this supplementation reversed the impaired intestinal sugar uptake observed in animals with DMH-induced pre-cancerous lesions, suggesting that it could be a good complementary therapy for the prevention of colon cancer in humans although more investigations are needed.

## Data Availability

The datasets generated during and/or analyzed during the current study are available from the corresponding author on reasonable request.
